# Evaluation of two endometriosis models by transplantation of human endometrial tissue fragments and human endometrial mesenchymal cells 

**Published:** 2017-01

**Authors:** Mina Jafarabadi, Mojdeh Salehnia, Rana Sadafi

**Affiliations:** 1 *Reproductive Health Research Center, Tehran University of Medical Sciences, Tehran, Iran.*; 2 *Department of Anatomical Sciences, Faculty of Medical Sciences, Tarbiat Modares University, Tehran, Iran.*

**Keywords:** Endometriosis, Matrix metalloproteinase 2, Osteopontin, Stromal cells

## Abstract

**Background:**

The animal models of endometriosis could be a valuable alternative tool for clarifying the etiology of endometriosis.

**Objective:**

In this study two endometriosis models at the morphological and molecular levels was evaluated and compared.

**Materials and Methods:**

The human endometrial tissues were cut into small fragments then they were randomly considered for transplantation into γ irradiated mice as model A; or they were isolated and cultured up to fourth passages. 2×10^6^ cultured stromal cells were transplanted into γ irradiated mice subcutaneously as model B. twenty days later the ectopic tissues in both models were studied morphologically by Periodic acid-Schiff and hematoxylin and eosin staining. The expression of osteopontin (OPN) and matrix metalloproteinase 2 (MMP2) genes were also assessed using real time RT-PCR. 17-β estradiol levels of mice sera were compared before and after transplantation.

**Results:**

The endometrial like glands and stromal cells were formed in the implanted subcutaneous tissue of both endometriosis models. The gland sections per cubic millimeter, the expression of OPN and MMP2 genes and the level of 17-β estradiol were higher in model B than model A (p=0.03).

**Conclusion:**

Our observation demonstrated that endometrial mesenchymal stromal cells showed more efficiency to establish endometriosis model than human endometrial tissue fragments.

## Introduction

Endometriosis (EMs) is a common gynecological disorder, which is characterized by abdominal pain, dysmenorrhea, infertility and irregular menstruation (1). It is an estrogen-dependent disease and recognized by the presence of endometrial tissue including endometrial glands and stromal cells outside the uterus (2). Some methods were proposed for treatment of EMs; however its pathogenesis has not yet been fully understood (3-6). Several studies have been focused on the establishment of endometriosis models, and showed that human endometrial tissue could be successfully transplanted into ectopic sites of animal models (7-11).

In homologous models of endometriosis, the endometrial tissue is removed and cut into small pieces then reintroduced into the peritoneal cavity of the same animal, whereas heterologous model has been developed by implanting human endometrial fragments from different phases of the menstrual cycle into immunodeficient mice (7, 8). The recovered transplanted tissue forms endometriotic-like lesions which their macroscopic and histological appearance were similar to those found in endometriosis patients. Heterologous models are very useful for understanding the mechanisms involved in the establishment of endometriosis as well as therapeutic testing of pharmacological and hormonal modulations (10-12). Pelch *et al* reported that the mouse model of surgically-induced endometriosis appears to be a good model for studying the pathophysiology and treatment of endometriosis (13).

The transplanted endometrial tissue has the ability of attachment and invasion to the host tissue through involvement of some intrinsic factors (14-17). Adhesion and invasion of endometrial tissue contribute both cell-to-cell and cell-to-matrix interactions which are mediated by adhesive molecules such as osteopontin (OPN) and proteolytic enzymes including matrix metalloproteinase (MMPs) (18, 19). OPN and MMPs are glycoproteins that are expressed in the human endometrium during implantation window as the best-characterized endometrial receptivity biomarkers (20, 21). OPN, a 70-kDa secretory glycoprotein, is mainly involved in cell adhesion and migration and binds to various integrin molecules as its receptors (18, 19, 22). 

The MMPs are belong to a family of proteins called the zinc metalloproteinase and involved in the endometrial shedding during menstruation and tumor invasion (23-25). The expression of MMPs in human endometrial tissue changes during different phases of menstrual cycle (26). Moreover several authors have studied the endometrial expression of OPN and MMPs in endometriosis patients with controversial results (27-30). One of the suggested mechanisms for impaired implantation in endometriosis patients is that abnormal expression of OPN and MMPs by eutopic endometrial tissues (31).

The existence of adult stem cells in endometrial tissue has been proved and these stem cells can be identified by the clonogenicity property, side population and expression of stem cell markers (32-34). It is proposed that endometrial stem cells are involved in etiology of endometriosis (35-37). In agreement with this hypothesis, there are some reports which demonstrated the potential of endometrial stem cells to produce endometriosis lesion after transplantation under kidney capsules of immunodeficient mice (38, 39).

The animal models of endometriosis could be a valuable alternative tool for clarifying the etiology of endometriosis (10). According to the best of our knowledge, there is a few information regarding to the establishment of endometriosis model using ectopic transplantation of endometrial stromal cell. There are some similarities between the endometrial stem cells and mesenchymal stem cells of other tissues like bone marrow (40), thus in the present study we mainly focused on the CD90 positive cells as a known mesenchymal stem cell marker. CD90 was used as a marker to identify the mesenchymal stem cells within the endometrial tissues by others (41-44). The immunophenotypes of these endometrial cells were analyzed using flow cytometry and their ability to differentiation to adipogenic and osteogenic, neural progenitors and glial-like cells were reported previously by our group (45, 46).

Thus the aim of the present study was to compare two endometriosis models by transplantation of human endometrial mesenchymal cells (CD90+) in comparison with human endometrial fragments to γ irradiated mice. These two models were evaluated by analysis of OPN and MMP2 genes expression in ectopic lesion and level 17-β estradiol in sera of mice.

## Materials and methods

Unless otherwise stated, all the chemicals and reagents used for this study were purchased from Sigma Aldrich (Germany).

**Human endometrial samples**

In this experimental study, the human endometrial samples from non-pathological women with aged 30-45 years (n=6) were obtained. These women had not used any hormonal treatment for three months prior to tissue removal. The tissues were transported to laboratory in Dulbecco Modified Eagle Medium /Hams F-12 (DMEM/F-12; Invitrogen, UK) containing 100 µg/ml penicillin G sodium, 100 µg/ml streptomycin sulphate B (Invitrogen, UK) and 5% fetal bovine serum (FBS; Invitrogen, UK) at 4^o^C.

Then, they were cut into small fragments (2×2×2 mm^3^) and considered randomly for further analysis. One fragment of each biopsy randomly was fixed in 4% buffered formaldehyde and embedded in paraffin wax for histological study. The normal morphology of the tissue was evaluated under light microscopy by experienced histopathologist. The samples in the proliferative phase had chosen for all experiments. Some fragments of each biopsy were considered for isolation and culturing of endometrial cells and the remaining tissue fragments were used for transplantation and molecular analysis. 

**Isolation and culture of human endometrial stromal cells**

The preparation of endometrial cells was according to the method of Gargett *et al* (44). Endometrial tissue (n=6 biopsy samples) was washed three times with phosphate buffered saline (PBS), transferred to DMEM supplemented with 10% FBS, and cut into 1-2 mm^3^ fragments for each sample. 

The specimens were digested and dissociated into single-cell using 300 µg/ml collagenase type 3, 40 µg/ml deoxyribonuclease type I (DNAase I) for 60-90 min at 37^o^C in 5% CO_2_. Then, the tissue was filtered through sterile meshes of 150, 100, 40 sieve (BD Biosciences) respectively (45). The collected endometrial stromal cells were resuspended with DMEM/F12 with10% FBS, and cultured in a six-well plate at 37^o^C in 5% CO_2_ and the media was changed every 2-3 days. 

**Flow cytometric analysis of cultured human endometrial stromal cells**

The immunophenotype of cultured endometrial stromal cells at fourth passage were assessed by CD90 marker as mesenchymal stem cell markers using flow cytometric analysis. Cultured endometrial cells trypsinized and plated at a density of 2×10^5^ cells/cm^2^ in DMEM medium containing 5% FBS. The cells were resuspended in 100 μl of PBS and incubated with direct APC-conjugated antibody (CD90) (1:100 dilutions) at 4^o^C for 1 hr. The labeled cells were washed in 100 μl of PBS and examined with a Flow Cytometric Calibur apparatus (FACS; Becton Dickinson). 

**Preparation of γ-irradiated mice**

Female National Medical Research Institute (NMRI) mice (6-8-weeks-old) with 20±1 gr weight were kept in university laboratory animal's house. The mice were treated with a single dose of 7.5 Gy γ-irradiation for 6 min 72 hr later the tissue and cells transplantation were performed in model A and B of endometriosis respectively (47).

The mice at prostrous phase were considered for transplantation (n=16 for all groups of study). Prior to transplantation, vaginal smears were prepared to prove the phases of mouse estrus cycle. The mice were anesthetized by an intraperitoneal injection of a mixture of 75 mg/kg body weight ketamine 10% and 15mg/kg xylazine 2% before transplantation under sterile conditions. All housing materials, as well as food and water, were autoclaved prior to use.

**Transplantation of endometrial tissue and mesenchymal stem cells to mice**

The mice at prostrous phase (n=6 for each group) were considered for transplantation in two groups of study as follows: In model A) the mice were received 3 pieces of endometrial tissue fragments (1-2 mm^3^) subcutaneously on one side of gluteal region via a dorso-horizontal incision then the wound was closed. In model B) the endometrial cells were harvested by 0.05% trypsin digestion and washed three times with PBS at the end of fourth passage. 

Approximately 20 µL of endometrial cell suspension which containing 2×10^6^ cells was injected by an insulin syringe subcutaneously on the one side of gluteal region via a dorso-horizontal incision then the wound was closed (48). Transplanted mice were maintained under sterile conditions for 20 days. Then the mice were sacrificed and the endometrial lesions were collected and randomly fixed for light microscopic study or kept at -80ºC for molecular analysis. 

**Light Microscopic study**

Human endometrial fragments before transplantation as control (n=6) and tissue lesion in two endometriosis models (n=3 for each group) were fixed in 10% formalin and embedded in paraffin wax. Tissue sections were prepared serially at 5 μm thickness and every fifth section of each sample was mounted on a glass slide and stained with hematoxylin and eosin and observed under light microscope with 100 magnification. In each tissue section, three fields of microscope were considered for evaluation of gland sections. 

The photos of each section were prepared and imported into Image J software. The area of each section was measured in units of pixels and converted to millimeters based on the conversion determined by measuring the image of the calibrated millimeter then the mean number of glands per mm^2^ was counted in each sample. For study of gland secretion another set of paraffin embedded tissue sections was prepared and mounted on a glass slide and stained with Periodic acid-Schiff (PAS) reaction and observed under the light microscope. 

**Hormonal assay**

Prior to mouse tissue sampling at prostrous phase, the blood samples were collected from the heart apex of mice then their sera were separated and kept at -20^o^C until hormonal analysis (n=6 for each model). The sera of non-transplanted mice (n=4) at prostrous phase were collected and considered as control. The concentrations of 17-β estradiol of sera were measured by an enzyme-linked immunosorbent assay kit (ELISA; Monobind, USA, sensitivity=6.5 pg/ml).

**RNA extraction and cDNA synthesis**

The mRNA levels of two endometriosis-related genes including OPN and MMP2 to housekeeping gene (GAPDH) were analyzed and compared in normal human endometrial tissue (before transplantation as control 1), human endometrial stromal cells (control 2) and endometriosis lesions derived from two endometriosis models (n=3 in each model).

RNA extraction was performed using an RNeasy MiniKit (Qiagen, Valencia, CA, USA), according to the manufacturer’s instructions. Using oligo dT, RNA was reverse-transcribed by Revert Aid M-MuL V reverse transcriptase using speciﬁed primers. Primers were synthesized based on human mRNA coding sequences. GAPDH gene was used as an internal control. The primers for real-time RT-PCR were designed using GenBank (http://www.ncbi.nlm.nih.gov) and Allele ID software (Table I) and ordered and synthesized by CinnaGen Co. (Iran, Tehran). 

**Real Time RT-PCR**

The real time RT-PCR was done by QuantiTect SYBR Green RT-PCR kit (Applied Biosystems, UK) after CDNA synthesis. The GAPDH was considered as reference gene and were ampliﬁed with the target genes in the same run. Real time thermal condition was including holding step: 95^o^C, 5', cycling step: 95^o^C 15', 58^o^C 30', 72^o^C 30' and it was continued by a melt curve step: 95^o^C 15', 60^o^C 1', 95^o^C 15'. Then the expression of the genes were analyzed by Pfaffel method. These experiments were done at least in three times. 

**Ethical consideration**

This study was approved by the ethics committee of medical faculty of Tarbiat Modares University (Ref no=52/11224). Informed written consent was obtained from each woman.

**Statistical analysis**

Statistical analysis was done with SPSS software (Statistical Package for the Social Sciences, version 19.0, SPSS Inc, Chicago, Illinois, USA). Quantitative variables were expressed as means±SD. The mean number of glands per mm^2^ and the results of the real time RT-PCR were compared by one-way ANOVA and the post hoc Tukey test. Mean hormone levels were compared using independent samples student’s t-test. P≤0.05 was considered significant.

## Results

**The morphology of cultured human endometrial cells**

**Figure 1:**
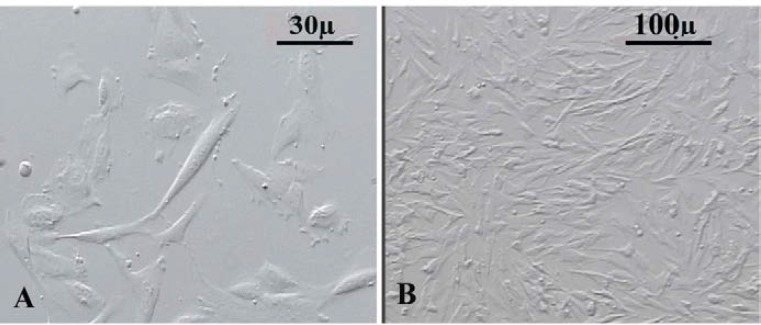
Phase contrast imaging of cultured human endometrial cells at first passage (A) and fourth passage (B).

After 24 hr some cells were adhered to the floor of plate and the population of these cells were heterogeneous in appearance (Figure 1A). After 3-4 passages, these cells became morphologically similar (Figure 1B).

**The proportion of CD90+ endometrial stromal cells using flow cytometry**

**Figure 2:**
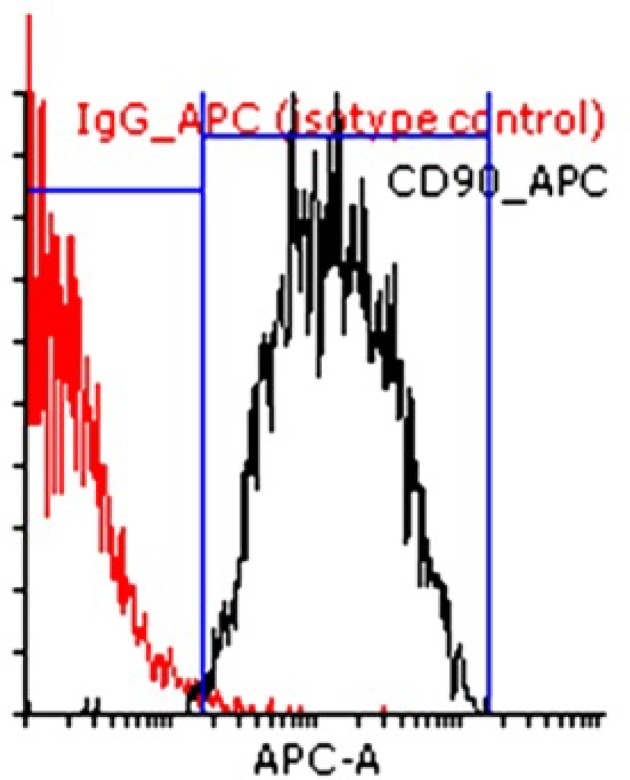
Flow cytometric analysis of CD90+ cultured endometrial stromal cells at the end of fourth passage. The histogram is the representative of three independent experiments. The red line shows the isotype control.

The immunophenotype of cultured endometrial stromal cells at the end of fourth passage revealed that the rates of CD90+ cells was 94.98±3 (Figure 2) in three repetitions. 

**The gross morphology of endometriosis lesion**

**Figure 3:**
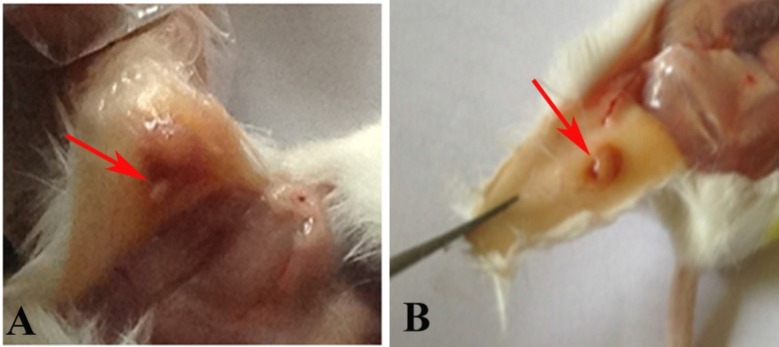
The gross morphology of endometriosis lesions in model A (A) and model B (B). As the arrow shows the lesions have cystic morphology

The macroscopic observation of endometriosis lesions in two models were shown in the Figure 3. As the figure showed. these lesions had cystic morphology and clearly was distinguished from the surrounding tissues. 

**Light microscopy observation**

**Figure 4:**
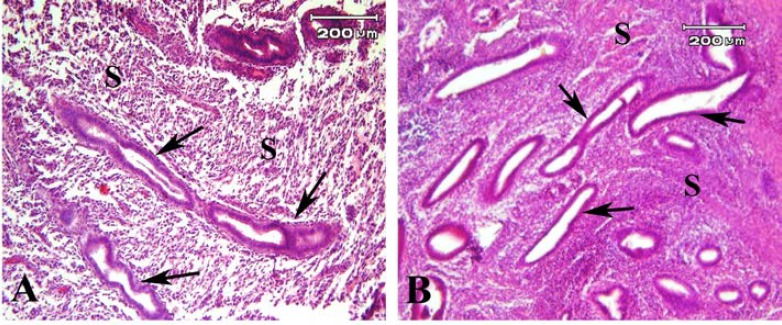
Light microscopy of normal human endometrial tissue were observed (A &B), the endometrial glands (black arrow) with stromal tissue (S) were seen

**Figure 5:**
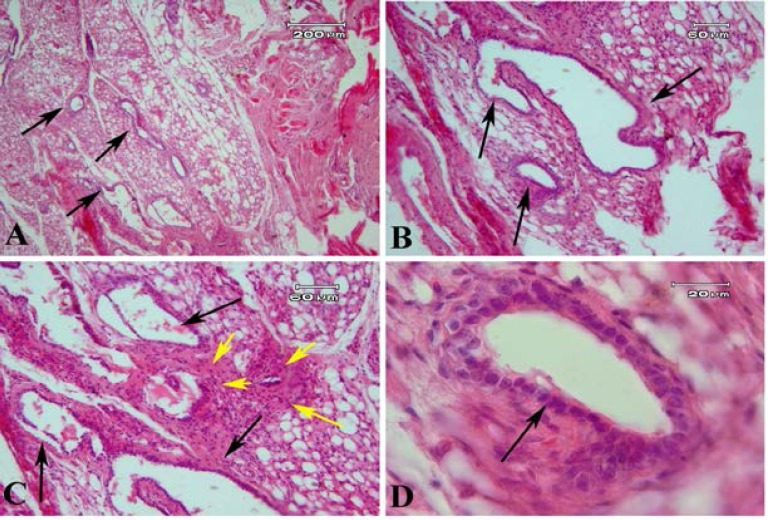
The morphology of recovered endometriosis lesion obtained from γ-irradiated mice stained by hematoxylin and eosin in model A. The black arrows indicate the endometrial like glands within the subcutaneous tissue of mice with low (A) and high magnification (B-D). The yellow arrows show the stromal cells between glands like sections

The morphology of human endometrial tissue before transplantation was normal and consists of a single layer of columnar epithelium at the surface and glandular portion (Figure 4 A, B). The representative light microscopic micrograph of transplanted endometrial tissue fragments which stained with H&E in model A is shown in Figure 5. The recovered endometriosis lesions firmly attached to underlying tissue. Photomicrograph of tissue sections of lesion demonstrated the presence of endometrial glands within the subcutaneous tissue. The flattened stromal cells with eosinophilic cytoplasm were seen between gland sections (Figure 5C).

**Figure 6:**
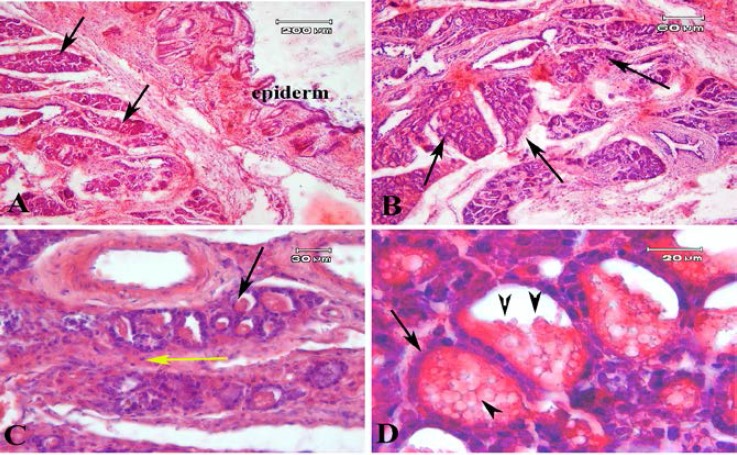
The representative photomicrographs of recovered endometriosis lesion stained by hematoxylin and eosin in model B. A lot of gland sections are seen within the subcutaneous tissue (black arrow) and these gland like sections are prominent in model B with low magnification (A & B) and higher magnification (C & D). Yellow arrow head shows the stromal cells between gland sections with high magnification (C). The black arrow head shows the eosinophilic secretion within these gland like structure (D).

**Figure 7:**
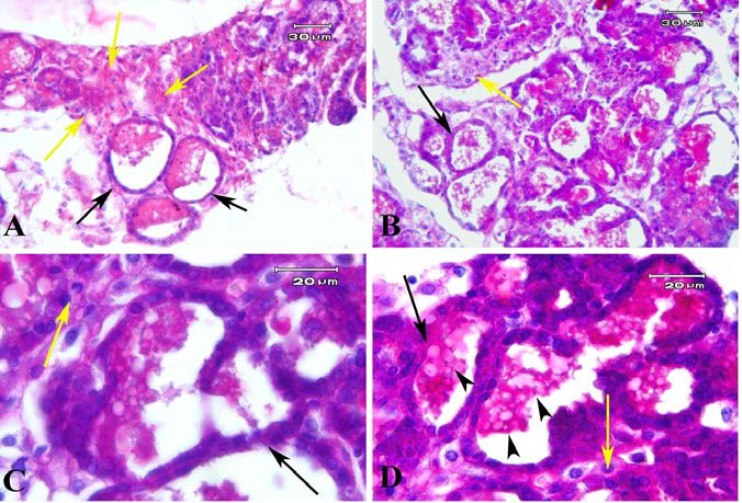
Photomicrograph of endometriosis lesions in model B stained with PAS method. It demonstrated the endometrial like glands which lined with cuboidal epithelial cells (black arrows) and PAS positive secretion within them (black arrow heads). The stromal cells between gland sections show the PAS positive reaction (yellow arrow

Some large cysts like glands which were lined by low columnar epithelial cells were seen (Figure 5 B, C). The tissue morphology of endometriosis lesion in model B were similar to model A and is presented in Figure 6. However, the endometrial like glands sections were prominent in subcutaneous tissue in model B compared to model A. These gland like structures were lined with cuboidal epithelium. Some eosinophilic secretions were seen within the lumen of these glands (Figure 6 C, D). The mean number of gland sections per mm^2^ in model A (57.55±17.18) was significantly lower than model B (271.57±77.98; p=0.03). The endometrial like gland structures within tissue sections of endometriosis lesions in both models showed positive reaction for Periodic acid-Schiff reaction (Figure 7). Also the stromal like cells within the gland sections showed the PAS positive reaction.

**Hormonal assay**

The level of 17-β estradiol in sera of mouse endometriosis model A and B and normal non-transplanted mice were 49.22±1.48, 63.32±1.71 and 40.05±0.83 pg/ml respectively (Table II). The level of 17-β estradiol was significantly increased in model B compared to model A and it was significantly higher in both models than the control group (p=0.03).

**OPN and MMP2 gene expression**

**Figure 8:**
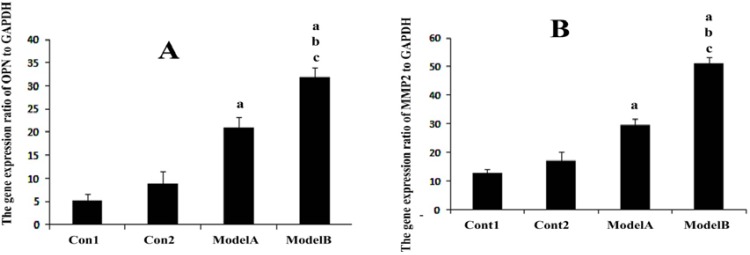
The expression ratio of OPN and MMP2 to GAPDH gene in normal endometrium as control1 and CD90+ mesenchymal stromal cells as control2 and in endometrial lesion in model A and model B. a: Significant differences with control1; b: Significant differences with control2; c: Statistically difference with model A (p=0.03

**Figure 9:**
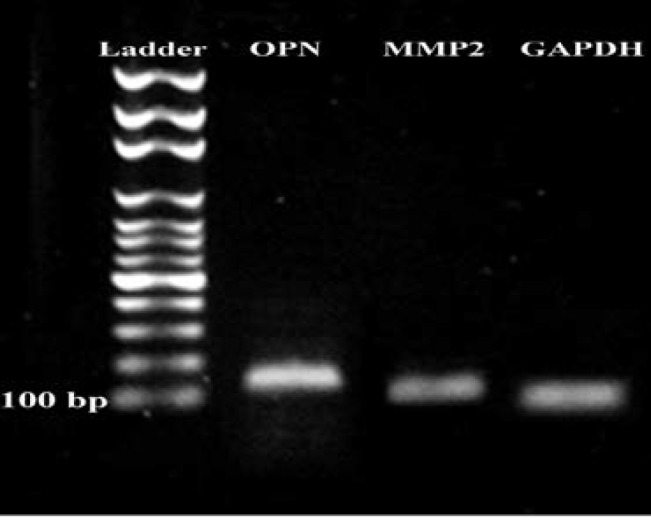
The gel electrophoresis of real time RT-PCR product. The ladder was 100bp. OPN: osteopontin; MMP2: metalloproteinase 2; GAPDH as internal control

The data regarding to gene expression are summarized and compared in Figure 8. The expression ratio of OPN and MMP2 to housekeeping gene in both recovered transplanted samples (endometriosis models A, B) was higher than the control 1 and control 2 (p=0.03) and these expression ratios in ectopic tissue in model B was higher than the model A (p=0.03). The gel electrophoresis of real time RT-PCR product is shown in Figure 9. 

**Table I:** Oligonucleotide primer sequences for real time RT-PCR

**Primer Name**	**CG%**	**Tm**	**Primer sequence**	**Length** **(bp)**
OPN (SPP1)F	55	58.1	5'- AGACCTGACATCCTGTACCC-3'	189
OPN (SPP1)R	57.89	58.4	5'- GTCGGTTTCAGCACTCTGG-3'	
MMP2F	55	57.5	5'- CGGCGAACCCATACTTCACA -3'	151
MMP2R	55	57.3	5'- GGCGAACGATACCCCTTTGA-3'	
GAPDH F	62.3	58	5'- CTGGGCTACACTGAGCACC-3'	101
GAPDH R	52.4	58	5'-AAGTGGTCGTTGAGGGCAATG-3'	

**Table ІІ:** The level of 17-β estradiol in sera of all studied groups

**Groups**	**Mean**	**± SD**
Control	40	3.83
Model A	49.22^a^	2.48
Model B	63.32^a^,^b^	3.27

a : significant differences with control (normal mouse sera) group (p<0.05) .

b : significant differences with Model A (p<0.05).

## Discussion

In this study we compared the potential of endometrial tissue fragments and isolated endometrial stromal cells to induce endometriosis lesions in mice. Our results demonstrated that in both models the endometrial like gland was formed. The PAS positive secretion was confirmed the nature of these glands which was similar to human endometriosis lesion. 

Moreover, this study, for the first time, showed that CD90+ endometrial cultured cells had the potential to create ectopic lesion of endometriosis after transplantation into mouse. The characteristics of these cells were demonstrated previously by our group using flow cytometric analysis (45, 46). These cells were positive for mesenchymal markers (CD90, CD105, CD146) and negative for epithelial (CD9) and hematopoietic (CD31, CD34) markers (45). The efficiency of gland formation in endometrial stromal cells induced model was better than the conventional model using fragment transplantation. One explanation for this observation may be the higher purification of mesenchymal CD90+ cells in cellular model (B). As their flow cytometric analysis confirmed that almost 95% of cultured cells at the end of fourth passage were CD90 positive. So it could be suggested that the proliferation of the mesenchymal stromal cells is involved in manifestation and pathogenesis of endometriosis. Other investigators also reported similar observation by transplantation of endometrial stem cells under kidney capsule of immunodeficient mice (38, 39).

We demonstrated that the level of 17-β estradiol was higher in both models compared to control group however model B produced significantly higher amounts. One explanation for this observation may be the increased glandular tissue formation in model B. Indeed, the estrogen dependence of endometriosis tissue was confirmed in several studies (2, 49). It was shown that the ectopic endometriotic tissue has high aromatase activity and this enzyme is responsible for the conversion of androstenedione and testosterone to estrone and estradiol. So the endometriosis implants can make estrogens from androgens by the aromatase enzyme (50). Our results confirmed the increased production of 17-β estradiol in both endometriosis models similar to human endometriosis patients. 

We also revealed that OPN and MMP2 mRNA levels were increased in endometriotic lesions in both endometriosis models in comparison with normal tissue however the expression of OPN and MMPs gene was higher in model B compared to the model A. Our findings on OPN gene expression are in agreement with other reports which have demonstrated an increase in OPN expression in a rat model of endometriosis in comparison to normal rats endometrial tissue and in patients with endometriosis in comparison with control subjects (28, 51-53). According to the functional role of OPN in cell adhesion, migration, differentiation and invasion of tumor cells, it could be assumed that OPN enhance endometrial invasiveness, proliferation and survival in ectopic lesions in animal models. Similar to our findings, Jiao *et al* using RT-PCR and western blotting, demonstrated that the expression levels of MMP2 and MMP9 were significantly increased in the rats endometriosis models compared to normal rats tissue (52). 

"Recently Malvezzi *et al* reported that the level of MMP2 is significantly higher in serum of infertile women with advanced stages of endometriosis (29)". "Pelch *et al* also showed that the expression of genes associated with the extracellular matrix, cell adhesions, immune function, cell growth, and angiogenesis are altered in the endometriotic lesion compared to the eutopic uterus (13)". An increased level of OPN and MMP mRNA in endometriosis models may be related to high concentration of estrogen. Wang and Ma evaluated the effects of estrogen and progestin on expression of MMP2 and tissue inhibitor of MMP2 in a nude mouse model of endometriosis and showed that estrogen can raise the expression level of MMP2 to promote ectopic implantation of endometrial tissue (43).

However, our findings support the idea that OPN and MMP may play a role in the pathogenesis of endometriosis, we suggest that it may be involved in attachment and invasion of the endometrium to ectopic sites. The presented model by our group shares some advantages described for the heterologous model using transplanting of human endometrial tissue into immunodeficient mice, such as availability and low costs, however, it also provides a promising tool not only for experimental approaches evaluating the etiology of endometriosis regarding to the type of responsible stem cells but also for therapeutic testing of pharmacological and hormonal modulations based on human endometrial stem cell however, judgment about its complete applications needs more investigations. 

## Conclusion

In conclusion, our observation demonstrated that endometrial CD90+ mesenchymal stromal cells showed more efficiency to establish endometriosis model human endometrial tissue fragments.

## Note

***This article extracted from M.Sc. thesis. (Rana Sadafi)***
